# Correlation of Physical Activity to Mental Health State and Grade Point Average Among Medical Students in Saudi Arabia: A Cross-Sectional Study

**DOI:** 10.7759/cureus.40253

**Published:** 2023-06-11

**Authors:** Yasser H Alnofaiey, Hashim M Atallah, Mohammed K Alrawqi, Hussam Alghamdi, Mohammed G Almalki, Jouman S Almaleky, Khalid F Almalki

**Affiliations:** 1 Department of Internal Medicine, College of Medicine, Taif University, Taif, SAU; 2 College of Medicine, Taif University, Taif, SAU

**Keywords:** grade point average, health-related effects of physical activity, depression, anxiety, academic achievement, students and academic achievement, physical activity level, physical activity

## Abstract

Introduction: Physical activity (PA) significantly impacts mental health. However, studies addressing the influence of PA on the mental health and academic performance of medical students are scarce.

Materials and methods: A cross-sectional study was carried out among 2,819 students pursuing their medical degrees in Saudi Arabia. PA was measured using the Global Physical Activity Questionnaire, and the state of their mental health was recorded using the Hospital Anxiety and Depression Scale. A multi-logistic regression was performed to predict the risk factor of poor academic performance.

Results: The prevalence of abnormal anxiety and depression levels were found to be 45.3% and 31.6%, respectively, which were comparatively more prevalent among students of younger age (p < 0.001). Students with abnormal anxiety levels had significantly lower grade point average (GPA) levels than others (p < 0.001). Those who were aged < 21 years, female gender, with chronic disease presence, low PA levels, and abnormal anxiety levels were independently associated with lower GPA levels (p < 0.05).

Conclusion: Low PA and high anxiety and depression levels were found to affect the academic performance of medical students in Saudi Arabia. Hence, health education about the importance of PA should be directed to those students.

## Introduction

Physical inactivity is considered the fourth leading cause of death globally [1]. According to the World Health Organization (WHO), physical activity (PA) is any skeletal muscle-driven movement involving energy use. All movement, whether done for recreation, transportation to and from locations, or as part of a person’s job, is considered physical exercise [2,3].

It is well known that regular engagement in any suitable form of PA (including walking, jogging, cycling, or swimming) has a positive physiological impact and lowers the risk of non-communicable diseases like cardiorespiratory disorders, type 2 diabetes, and hypertension, as well as other chronic diseases and comorbidities that can cause death [2]. Lack of PA has been related to low student achievement [3]. PA has been shown to have a favorable effect on cognitive performance, and adequate physical exercise helps in reducing stress and improving intellectual capabilities, enhancing students’ academic achievement. A certain level of PA is required for optimal mental function and mental concentration and helps manage stress, which gradually deteriorates academic achievement [4]. Adequate PA has been reported to improve the quality of life among students as it increases physical self-respect [5], improves self-concept and cognition, induces arousal, and reduces boredom and stress [6]. Advocating for increased physical exercise in schools and among the elderly has also been a popular strategy for preventing and addressing cognitive decline [6].

Previous studies have reported an association between a lack of PA and anxiety, depression, and poor mental health, and life quality [3,5]. To illustrate, medical students devote more time to their studies at the expense of PA, particularly if examinations are approaching [4]. Medical students experience many challenges (long-lasting medical education, encounters with suffering patients and death, and insufficient time), which impact the medical students’ quality of life [5]. This information confirms that these students are especially vulnerable to anxiety and depression, which is especially troublesome in the medical profession [4]. Studies involving medical students have revealed significant positive relationships between PA and academic performance and self-esteem [4,7]. In fact, a positive relationship was found between high academic achievement and performing physical exercise for at least 30 minutes per day for five days a week [6].

The WHO, the American College of Sports Medicine, and the American Heart Association state that all healthy adults between the ages of 18 and 65 years need to engage in moderate-intensity aerobic (endurance) PA for at least 30 minutes, five days per week or vigorous-intensity aerobic physical exercise for at least 20 minutes, three days per week, to promote and maintain health [8].

In the Kingdom of Saudi Arabia (KSA), a significant association between PA and academic performance has been reported [9]. Moreover, significant relationships were found among participants’ age, gender, body mass index (BMI), cortisol, serotonin, PA score, academic performance, and executive function [9]. However, in KSA, limited studies have explored the influence of PA on mental health and academic performance.

The main purpose of this study is to assess the association between PA and mental health status and academic achievement among medical students in Saudi Arabia.

## Materials and methods

This descriptive cross-sectional study was conducted on medical interns and undergraduate medical students from the 2nd year until the 6th year. The pre-medical year and 1st medical year students in Saudi Arabia were excluded. A total coverage was done, and all students in the selected academic years were contacted through emails from the Office of Student Affairs. Data were collected using a self-administered electronic questionnaire. A minimum sample size of 2,819 students was calculated with a 5% margin of error and a 95% confidence interval, incorporating the values from a previous study by Alghadir et al. [9]. The survey was conducted from November 2021 to March 2022.

The questionnaire was distributed to all participants and included three parts. The first part collected data about demographic characteristics, grade point average (GPA), which measures student performance in implementing the curriculum [6], and BMI. The second part collected details regarding PA, and the last assessed the participants’ mental health status.

Assessment of the study variables

The demographic data were the following: gender, age, marital status, chronic diseases, name of the university, BMI, and GPA. BMI was calculated by dividing weight in kilograms by height in meters squared and classified as underweight (BMI < 18.5 kg/m2), normal weight (18.5-24.9 kg/m2), overweight (25.0-29.9 kg/m2), or obese (30.0 kg/m2 or greater). The students’ GPA was used as a measure of academic achievement. The students were classified based on the system of the university either out of 5 or 4: excellent (4.5-5/3.5-4), very good (3.75-4.49/2.75-3.49), good (2.75-3.74/1.75-2.74), and passed (2-2.74/1-1.74).

PA was assessed by the Global Physical Activity Questionnaire (GPAQ), developed by the WHO for country-level PA surveillance, consisting of 16 questions that collect data on PA participation in three domains and sedentary behaviors. The domains are work activity, travel to and from places, and recreational activities. Based on the median total of the PA, the participants were classified as high, moderate, and low. In addition, the students were classified as having high, moderate, or low activity based on the metabolic equivalent of task (MET) [10]. Mental health state, including anxiety and depression, was assessed by the Hospital Anxiety and Depression Scale (HADS). Depression and anxiety were measured using the HADS questionnaire, a valuable tool utilized by many studies for this purpose. The HADS questionnaire was employed because it is a verified instrument that can assess depressive and anxiety symptoms in patients who appear to be in good health. An extensive sample of French employees from 16 major organizations was tested using the HADS in 2014, and it was validated to identify anxiety and depressive disorders [11]. The HADS questionnaire is a suitable measure for assessing anxiety and depression in medical students, according to a master’s thesis from Cardiff University’s Philosophy Department done in 2019 [12]. The HADS scale was found to have an optimal cut-off ≥ 8 (sensitivity of 0.80 and specificity of 0.88) [13].

The HADS is a 14-item scale with seven items each for the anxiety and depression subscales. The score for each item ranges in value from 0 to 3. A subscale score of 8 or above indicates anxiety or depression [14].

The Research Ethics Committee approved the present study in the College of Medicine of Taif University, Taif, Saudi Arabia, (approval number: 43-086; dated: 28/11/2021). All data were kept in a secured file and only accessed by the research team. This ensured the participants’ data will be used for research purposes only. All methods followed the relevant guidelines and regulations.

Data analysis

Data were analyzed using SPSS version 26 (IBM Corp., Armonk, NY). Qualitative data were expressed as numbers and percentages to test the relationship between variables, and the chi-square test (χ2) was used. Quantitative data were expressed as mean and standard deviation (mean ± SD). A multi-logistic regression model was done to assess the risk factors for poor academic performance. A p-value of less than 0.05 was considered statistically significant.

Availability of data and materials

All data analyzed during this study are included in this published article.

## Results

We received a total of 2,819 responses with the mean age of the participants being 22.17 ± 2.28 years. The socio-demographic analysis showed 66.5% were females, 24.2% were in the 5th academic year, 45.3% had a monthly family income of more than 15,000 Saudi riyals (SR), 48% had a GPA of 4.5-5/3.5-4 (out of 5 or 4), 11.1% were smokers, 9.8% had chronic diseases, and 51.7% had normal BMI. The mean HADS scores for anxiety and depression were 9.98 ± 3.12 and 8.68 ± 3.13, respectively (Table 1).

**Table 1:** Baseline characteristics of the students (n = 2,819) GPA: grade point average; BMI: body mass index; HADS: Hospital Anxiety and Depression Scale; SD: standard deviation.

Variable	No. (%)
Age	22.17 ± 2.28
Gender	
Female	1,875 (66.5)
Male	944 (33.5)
Academic year	
2nd	503 (17.8)
3rd	498 (17.7)
4th	491 (17.4)
5th	681 (24.2)
6th	451 (16)
Intern	195 (6.9)
Monthly income	
Less than 4,000	335 (11.9)
4,000–7,000	422 (15)
8,000–15,000	786 (27.9)
More than 15,000	1,276 (45.3)
GPA out of 5 or 4	
2-2.74 (out of 5)/1-1.74 (out of 4)	57 (2)
2.75-3.74 (out of 5)/1.75-2.74 (out of 4)	417 (14.8)
3.75-4.49 (out of 5)/2.75-3.49 (out of 4)	992 (35.2)
4.5-5 (out of 5)/3.5-4 (out of 4)	1,353 (48)
Smoking status	
Non-smoker	2,506 (88.9)
Smoker	313 (11.1)
Chronic diseases	
No	2,551 (90.5)
Yes	268 (9.5)
BMI categories	
Underweight	387 (13.7)
Normal weight	1,457 (51.7)
Overweight	638 (22.6)
Obese	337 (12)
BMI (mean & SD)	24.06 ± 8.22
HADS anxiety score (mean & SD)	9.98 ± 3.12
HADS depression score (mean & SD)	8.68 ± 3.13

**Figure 1:**
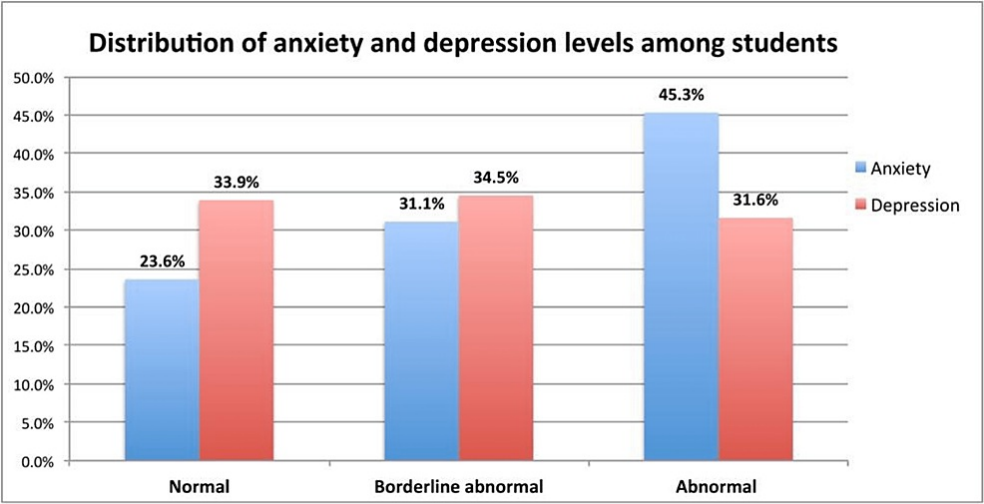
Distribution of anxiety and depression levels among students

**Figure 2:**
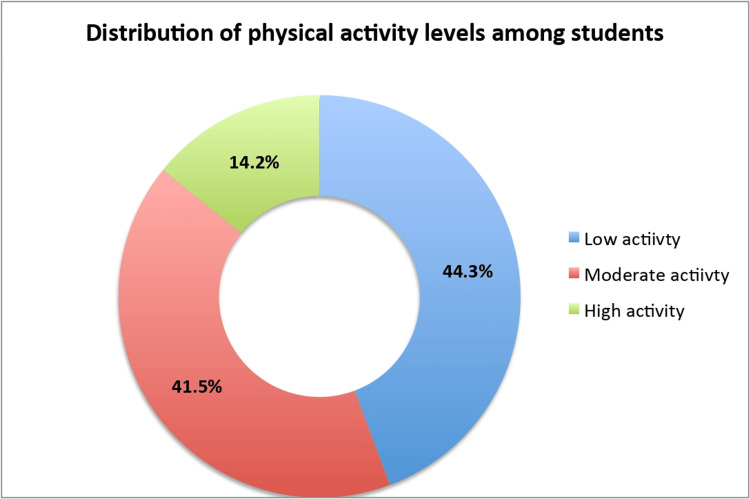
Distribution of physical activity levels among students

The prevalence of borderline abnormal and abnormal cases of anxiety among students was 31.1% and 45.3%, respectively (Figure 1). In comparison, the prevalence of borderline abnormal and abnormal cases of depression was 34.5% and 31.6%, respectively (Figure 1). The assessment of PA showed that only 14.2% had high PA levels while 41.5% and 44.3% had moderate and low PA levels, respectively (Figure 2).

The assessment of the relationship between mental health status and students’ baseline characteristics is given in Table 2. Both abnormal anxiety and depression cases were comparatively higher among students of younger age (p < 0.001). Female students demonstrated significantly more abnormal anxiety (p < 0.001) and depression (p < 0.001) cases compared to males. Students who reported a monthly income of <4,000 SR had a significantly higher prevalence of abnormal depression (p = 0.041). Additionally, the prevalence of abnormal anxiety levels was significantly higher among those with chronic diseases (p < 0.001). A non-significant relationship was found between anxiety level and students’ monthly income, smoking status, and BMI categories (p > 0.05). No significant association was observed between depression levels, students’ smoking status, and BMI categories (p > 0.05). Students who had abnormal anxiety levels had significantly low GPA levels compared to others (p < 0.001); whereas, there was no significant association observed between GPA and depression level (p = 0.557). It was observed that students who had low levels of PA had significantly more abnormal depression levels (p < 0.001) and abnormal anxiety levels (p < 0.001).

**Table 2:** Relationship of anxiety and depression with baseline characteristics of the participants BMI: body mass index; GPA: grade point average; SR: Saudi riyal.

	Anxiety				Depression			
Variables	Normal	Borderline abnormal	Abnormal	p-value	Normal	Borderline abnormal	Abnormal	p-value
Age (years)	22.45 ± 2.34	22.32 ± 1.99	21.92 ± 2.4	<0.001	22.4 ± 2.17	22.15 ± 2.43	21.95 ± 2.2	<0.001
Gender								
Female	374 (19.6)	562 (30)	939 (50.1)	<0.001	591 (31.5)	643 (34.3)	641 (34.2)	<0.001
Male	290 (30.7)	315 (33.4)	339 (35.9)		364 (38.6)	329 (34.9)	251 (26.6)	
Academic year								
2^nd^	82 (16.3)	119 (23.7)	302 (60)	<0.001	130 (25.8)	183 (36.4)	190 (37.8)	<0.001
3^rd^	112 (22.5)	149 (29.9)	237 (47.6)		159 (31.9)	167 (33.5)	172 (34.5)	
4^th^	99 (20.2)	149 (30.3)	243 (49.5)		147 (29.9)	167 (35.8)	168 (34.2)	
5^th^	176 (25.8)	243 (35.7)	262 (38.5)		256 (37.6)	230 (33.8)	195 (28.6)	
6^th^	130 (28.8)	149 (33)	172 (38.1)		178 (39.5)	151 (33.5)	122 (27.1)	
Intern	65 (33.3)	68 (34.9)	62 (31.8)		85 (43.6)-	65 (33.3)	45 (23.1)	
Monthly income								
<4,000 SR	74 (22.1)	96 (28.7)	165 (49.3)	0.067	93 (27.8)	121 (36.1)	121 (36.1)	0.041
4,000–7,000 SR	82 (19.4)	128 (30.3)	212 (50.2)		137 (32.5)	143 (33.9)	142 (33.6)	
8,000–15,000 SR	181 (23)	252 (32.1)	353 (44.9)		255 (32.4)	277 (35.2)	254 (32.3)	
>15,000 SR	327 (25.6)	401 (31.4)	548 (42.9)		470 (36.8)	431 (33.8)	375 (29.4)	
Smoking status								
Non-smoker	592 (23.6)	776 (31)	1138 (45.4)	0.893	859 (34.3)	870 (34.7)	777 (31)	0.115
Smoker	72 (23)	101 (32.3)	140 (44.7)		96 (30.7)	102 (32.6)	115 (36.7)	
Chronic diseases								
No	614 (24.1)	811 (31.8)	1126 (44.1)	<0.001	870 (34.1)	877 (34.4)	804 (31.5)	0.733
Yes	50 (18.7)	66 (24.6)	152 (56.7)		85 (31.7)	95 (35.4)	88 (32.8)	
BMI categories								
Underweight	82 (21.2)	112 (28.9)	193 (49.9)	0.084	125 (32.3)	136 (35.1)	126 (32.6)	0.754
Normal weight	331 (22.7)	446 (30.6)	680 (46.7)		507 (34.80	503 (34.5)	447 (30.7)	
Overweight	170 (26.6)	205 (32.1)	263 (41.2)		217 (34)	221 (34.6)	200 (31.3)	
Obese	81 (24)	114 (33.8)	142 (42.1)		106 (31.5)	112 (33.2)	119 (35.3)	
GPA								
2-2.74/1-1.74	7 (12.3)	10 (17.5)	40 (70.2)	<0.001	17 (29.8)	21 (36.8)	19 (33.3)	0.557
2.75-3.74/1.75-2.74	100 (24)	139 (33.3)	178 (42.7)		139 (33.3)	134 (32.1)	144 (34.5)	
3.75-4.49/2.75-3.49	271 (27.3)	312 (31.5)	409 (41.2)		355 (35.8)	334 (33.7)	303 (30.5)	
4.5-5/3.5-4	286 (21.1)	416 (30.7)	651 (48.1)		444 (32.8)	483 (35.7)	426 (31.5)	
Physical activity								
Low	268 (21.5)	395 (31.6)	585 (46.8)	<0.001	400 (32)	414 (33.1)	435 (34.8)	<0.001
Moderate	257 (21.9)	358 (30.6)	555 (47.4)		380 (32.4)	421 (36)	369 (31.5)	
High	139 (35)	123 (31)	138 (34)		176 (44)	136 (34)	88 (22)	

A logistic regression model was performed where a low GPA (2-2.74/1-1.74) was considered the dependent variable. It was found that age < 21 years (OR = 2.13; 0.82-4.50, p = 0.015), female gender (OR = 3.39; 1.03-5.21, p = 0.032), presence of chronic disease (OR = 1.81; 0.89-3.87, p = 0.026), low levels of PA (OR = 2.17; 0.91-6.65, p = 0.002), and abnormal anxiety levels (OR = 2.28; 1.03-3.98, p = 0.042) were independently associated with low GPA levels (Table 3).

**Table 3:** Logistic regression model BMI: body mass index; GPA: grade point average; SR: Saudi riyal.

Low GPA = dependent variable		Odds ratio (OR)		95% CI for OR				p-value
				Lower	Upper			
Age <21 years	2.13		0.82			4.50	0.015	
Gender = female	3.39		1.03			5.21	0.032	
Academic year = 2^nd^ year	0.74		0.21			2.56	0.632	
Monthly income =<4,000 SR	0.64		0.19			2.16	0.175	
Smoking	0.40		0.12			1.65	0.481	
Chronic diseases	1.81		0.89			3.87	0.026	
BMI >25	0.67		0.92			2.09	0.217	
Low physical activity	2.17		0.91			6.65	0.002	
Abnormal anxiety level	2.28		1.03			3.98	0.042	
Abnormal depression level	0.56		1.82			4.72	0.187	
Constant	0.038		Non			Non	0.006	

## Discussion

The major goal of this study was to evaluate the relationship between PA, mental health state, and GPA among Saudi medical students. This study demonstrated that low PA levels were significantly associated with depression and anxiety in these medical students. In non-clinical and clinical populations, the psychological state contributes to the individuals’ total and subjective well-being [15]. Practicing PA was associated with better mental health in children and adolescents, but many studies had inadequate designs, which led to minor to moderate impacts on mental health. The main strength of this study was that it used a comparatively larger sample to look at the demographic and the other factors that impact students’ mental health. The WHO and American College of Sports Medicine recommend that healthy adults indulge in 30 minutes of moderate-intensity PA (five days a week, % of VO2 max) or 20 minutes of vigorous-intensity PA (three days a week, >75% VO2 max > 6 METs) per day, five days per week, to maintain or improve their health. Additional workouts have been advised to further lower the risk of obesity and chronic diseases such as diabetes, hypertension, and cardiovascular events [16,17].

Students at the university level report experiencing significant levels of perceived stress and cognitive workload, and recent evidence indicates that student-counseling services are seeing an increase in the number of health professional students seeking assistance [18]. According to survey reports from different continents, one out of every five university students reports having depressive symptoms or a moderate to severe form of depression [19-21]. In the findings of this study, females demonstrated significantly higher depression and anxiety levels than male students. The findings correlated with prior research in different contexts [22-24]. These differences could be explained based on the physiological differences between females and males (e.g., genetic susceptibility and hormonal variations) that could influence mental and behavioral patterns [25]. Females are more likely to feel anxious and suffering; thus, females might be more exposed to depression and anxiety than men [26]. The correlational findings in this study between PA and mental health cannot be treated as evidence of causal relationships, but the aforementioned study findings indicate that regular PA protects university students from mental health problems [27]. This study’s findings showed a significant association between a lower GPA and higher anxiety levels, but no significant association was observed between lesser PA and higher depression levels. A study by Al-Drees et al. in Riyadh province, Saudi Arabia, reported that 47.2% of the students were practicing PA, and these students had higher GPA levels than inactive students [6]. Previous studies have indicated positive associations between PA and academic achievement [28-30]. Reduced PA activity leads to weight gain, which could affect medical students’ cognitive functions [31]. Short-term PA habits may have beneficial effects on mental health and physical well-being, which may help students improve their cognitive functioning.

Moreover, exercise is reported to increase grey and white matter volume, thereby improving cognitive and central nervous system functioning [32]. It has been observed that the anxiety and depression levels were significantly higher among the junior students (2nd and 3rd years) than senior students. This could be explained by the fact that these junior students are transitioning during these academic years and this may be a particularly vulnerable period of adjustment [21]. Evidence also suggests that first-year undergraduates are more prone than final-year graduate or postgraduate students to suffer from some forms of depression [33]. It is reported that peer social support, helpful friendships, encouragement, and a supportive academic environment could improve PA habits among students [33]. Based on the overall findings of this study, raising medical students’ awareness about the importance of PA through health education campaigns is recommended, with an emphasis on the association between PA and academic achievement.

Limitations

This study used a self-reported questionnaire that could have a recall bias. In addition, having a cross-sectional study design could reveal the associations between variables without addressing the casual relationships. Also, carrying out the study only on medical students could affect the generalizability of results on all Saudi university students. In addition, factors such as smoking, drug use, menstrual dysphoric syndrome, and family history of mental disorders were not included in the study, which could affect the study’s results.

## Conclusions

The study revealed that low PA, higher anxiety, and depression affected the academic performance of medical students in Saudi Arabia. Students who were younger than 21 years, females, with chronic diseases, low PA, and abnormal anxiety levels were independent predictors of low GPA levels. This study contributes to the PA, mental health, and GPA literature using a cross-sectional convenience sample. Additional research is needed to prove the absence of a link between PA, mental health, and GPA. With over six years of education, it is also vital to investigate among medical students the longitudinal relationships between PA and its relationship with their academic performance.

## Figures and Tables

**Figure 3:**
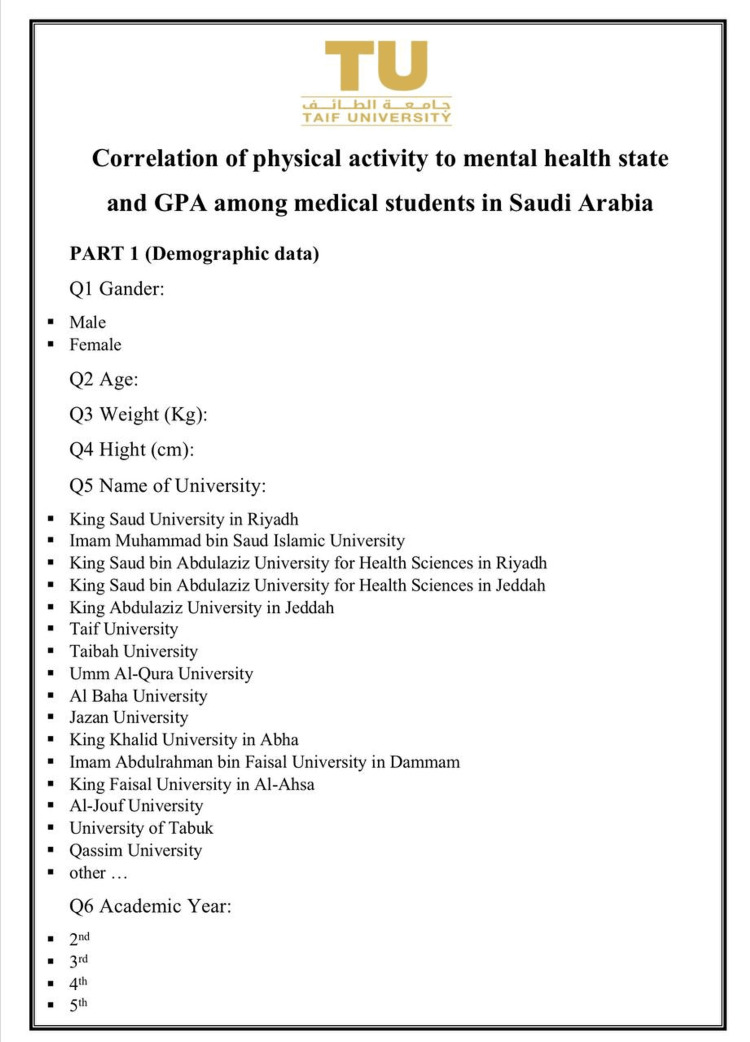
Questionnaire page 1

**Figure 4:**
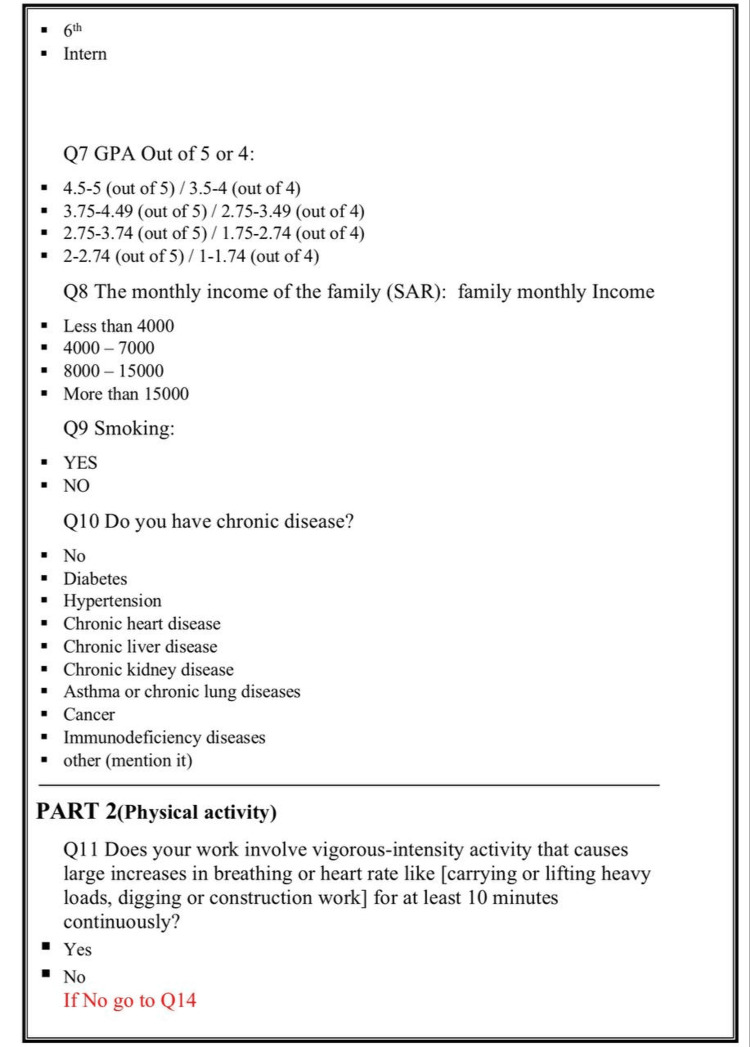
Questionnaire page 2

**Figure 5:**
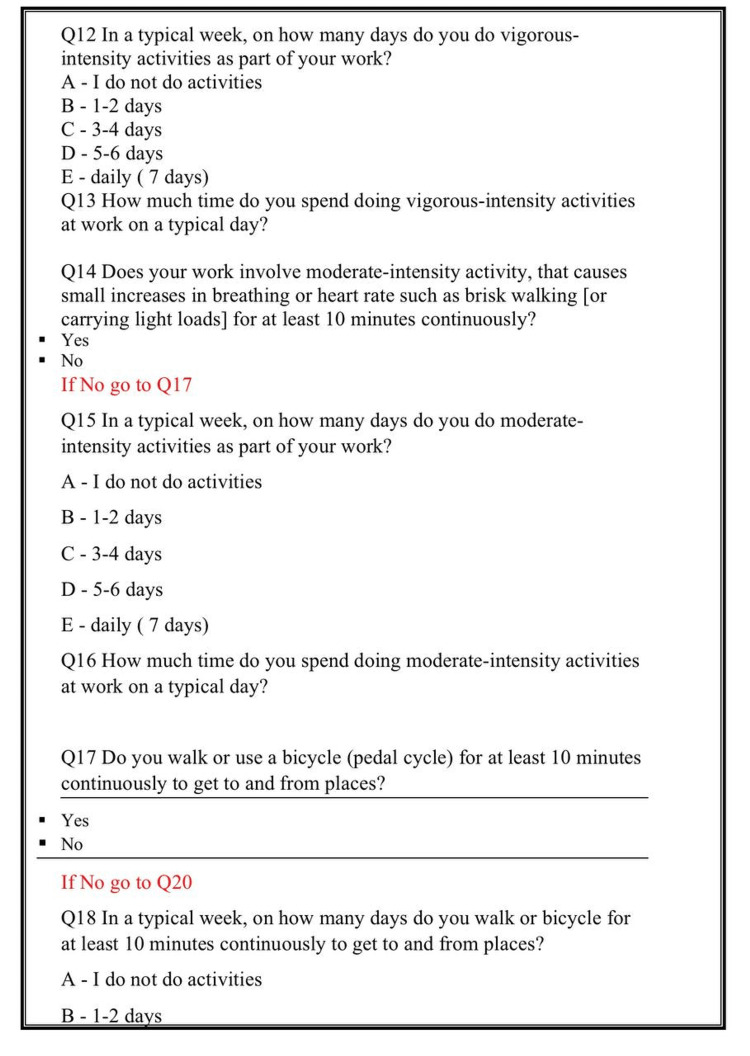
Questionnaire page 3

**Figure 6:**
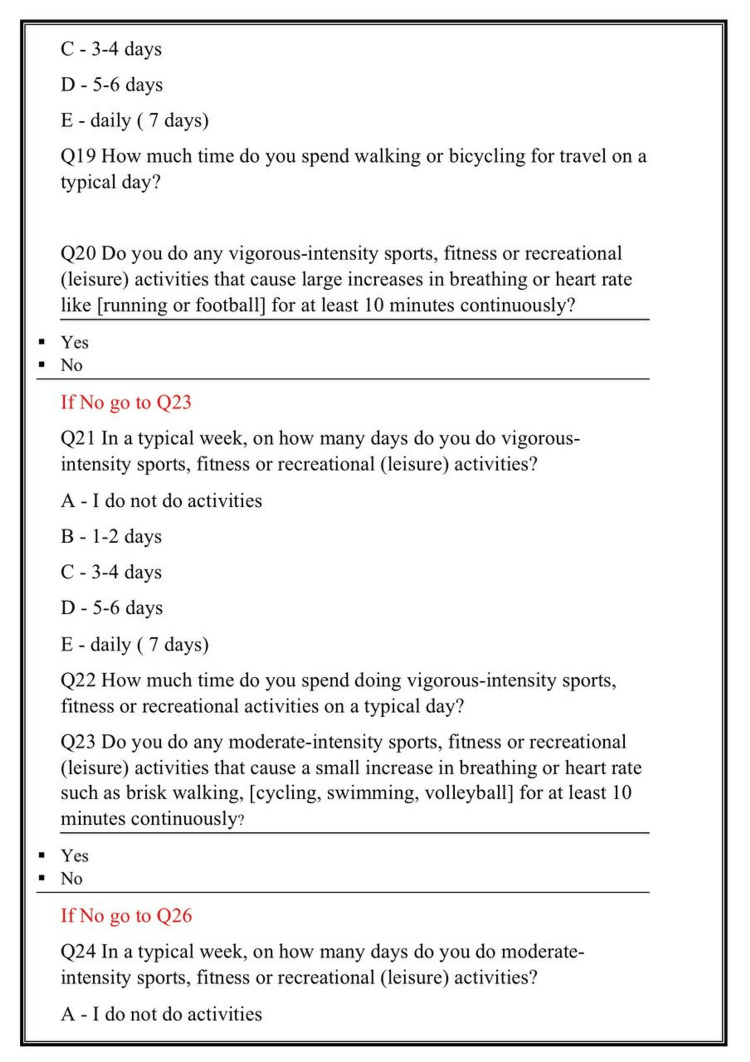
Questionnaire page 4

**Figure 7:**
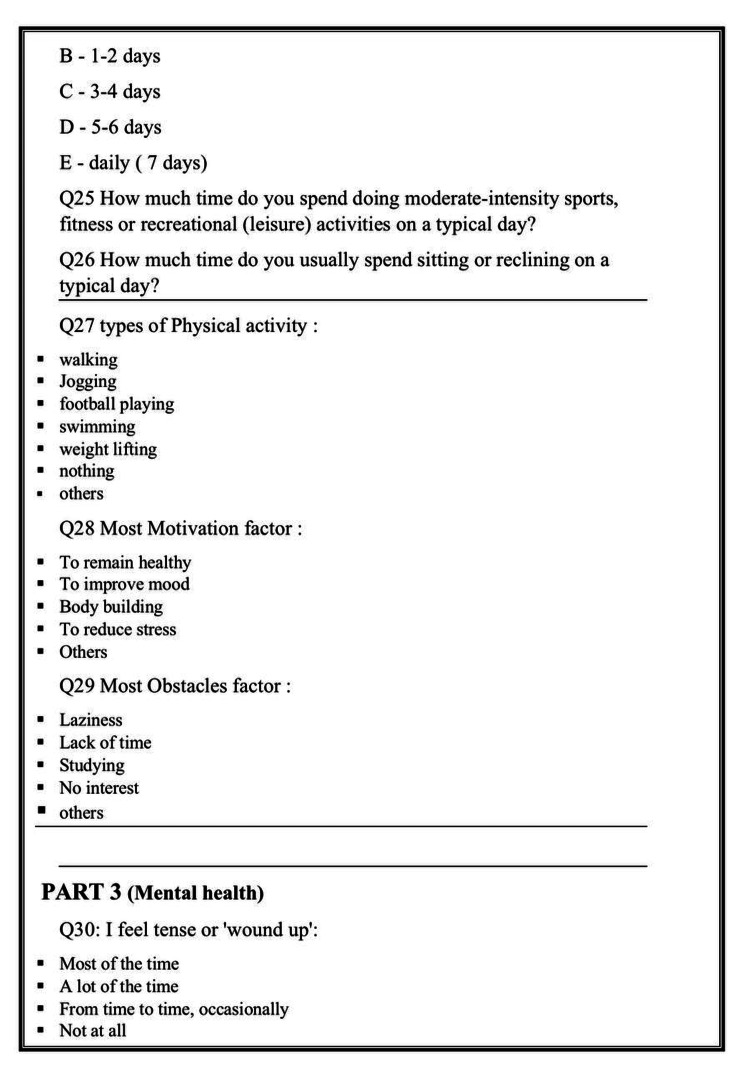
Questionnaire page 5

**Figure 8:**
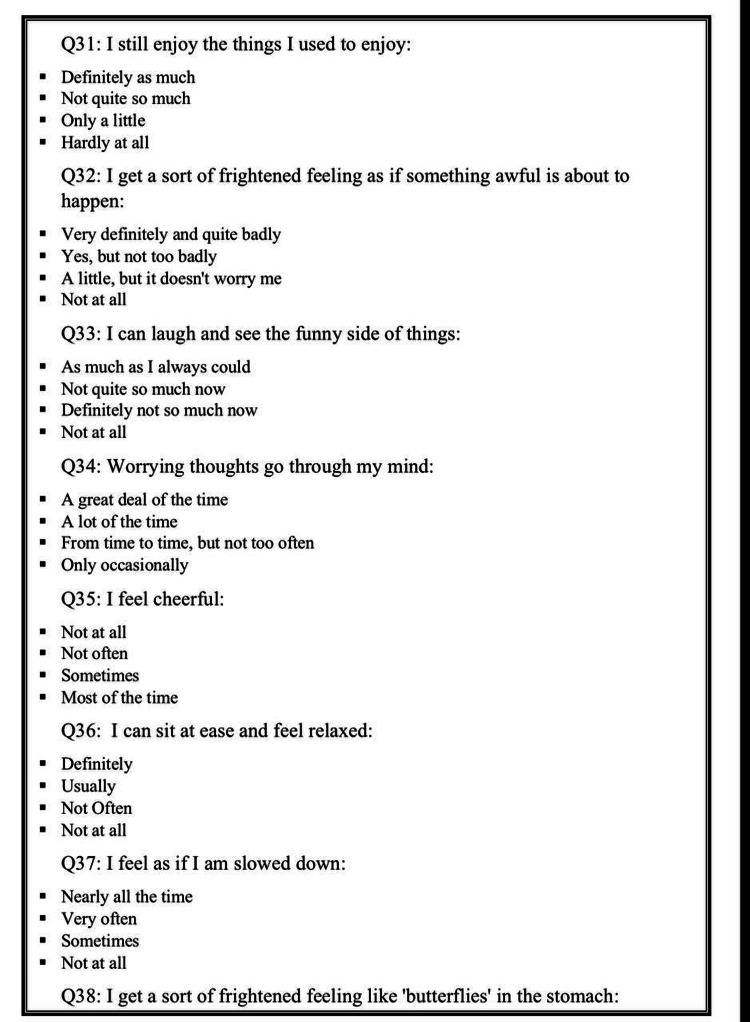
Questionnaire page 6

**Figure 9:**
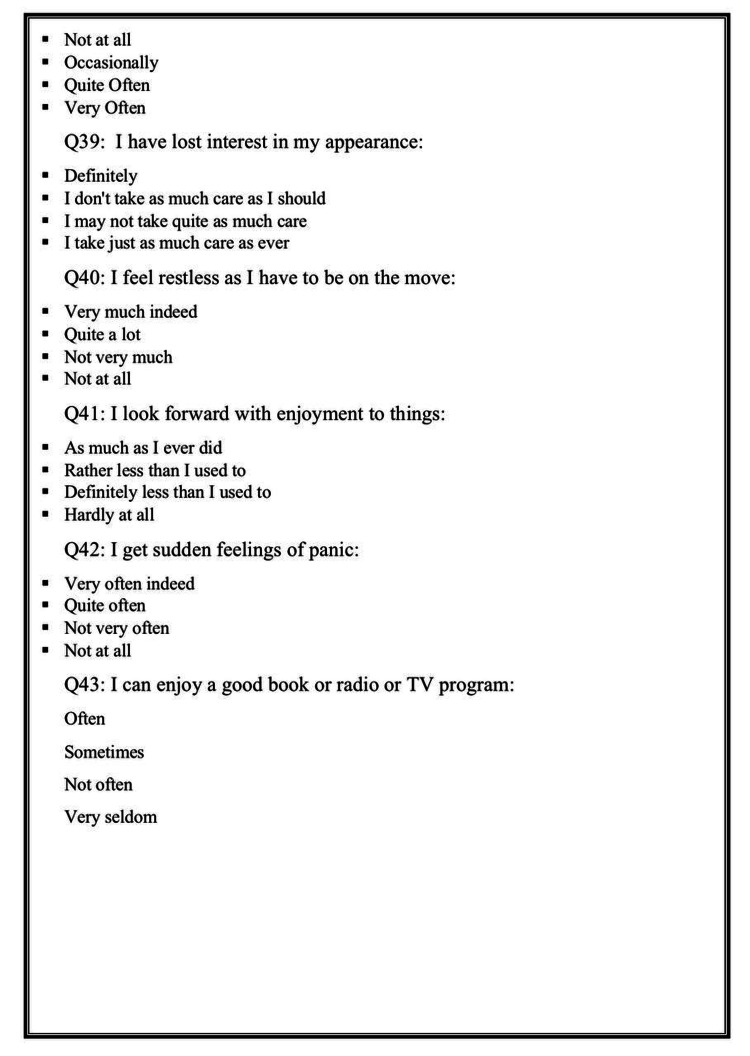
Questionnaire page 7

## Appendices

Questionnaire
